# Neuropsychiatric Alterations in a Patient Diagnosed with Advanced Korsakoff's Syndrome: Clinical Case of Low Incidence and Prevalence in Colombia

**DOI:** 10.1155/2022/2772594

**Published:** 2022-12-31

**Authors:** Carlos Alberto Hurtado Gonzalez, Sebastian Ospina Otalvaro, Carlos Steven Marmolejo Escobar, Karen Julieth Quebrada Mera, Pablo Miguel Arango de la Pava, Carlos Andres Clavijo, Lucely Ortega Bolaños, Narda Rátiva Hernández, Angelica Maria Vidal Rosero, Paola Andrea Gutierrez Lenis, Armando Lucumí, Jean Paul Cappellaro Sánchez, Angela Agudelo Zamorano, Juan Pablo Jacome, Valentina Herrera Montoya, Luis Miguel Saldarriaga, Carolina Prado Salcedo, Sharon Fabiana Alvarado Carranza, Carlos Andres Marín Hoyos, Juan Pablo Beltran Alomia, Juan Felipe Ayala Rico, Luigui Andres Torres Colorado, Julia Andrea Arias Díaz

**Affiliations:** ^1^Research Department, Clinical Neuropsychology, Research Team SEMINEC Cooperativa University, Cali, Colombia; ^2^Research Department, Department of Medicine and Psychiatry, Research Team Microambiente, Libre University, Cali, Colombia; ^3^Neurosurgeon Department of Medicine, Del Valle University, Cali, Colombia; ^4^Neurology, Neurological Institute of the Pacific, Cali, Colombia; ^5^The National Autonomous University of Mexico UNAM, Mexico City, Mexico; ^6^Specialty of Psychiatry, Libre University, Cali, Colombia; ^7^Physical Therapy Program, Faculty of Health and Sports Sciences, University Foundation of the Andean Area, Bogota, Colombia

## Abstract

Korsakoff's syndrome (KS) is an insidious and progressive neuropsychiatric disorder that affects specific neurocognitive functioning, especially in tasks that require sustained attention, memory, executive functions, and visuospatial functioning. Usually, this disease generates neuropsychiatric complications that worsen the quality of life (QOL) of patients in the medium term. We present a case of a 63-year-old male who presented with a diagnosis of advanced Korsakoff's syndrome and has a clinical history of recurrent memory loss and a history of alcohol abuse. The patient showed difficulty manipulating immediate information, associated with a possible frontal lobe dysfunction, and inability to remember material given through the auditory pathway. The patient showed a psychiatric clinical picture which is constantly worsening his and his immediate caregiver's QOL. The data obtained demonstrate that the patient presents a progressive cognitive impairment, which in the short term is correlated with Korsakoff-type dementia. It is suggested to carry out functional neurorehabilitation plans aimed at improving the QOL of the patient, his immediate caregiver, and future people with this type of diagnosis.

## 1. Introduction

Korsakoff's syndrome (KS) is a neuropsychiatric disease, also defined like a rest state after the encephalopathic disease phase of Wernicke's encephalopathy. KS is caused by the loss of vitamin B1 [1]. Its symptoms include the inability of the patient to form memories related to his immediate verbal memory and alterations in executive function tasks, especially in inhibition tasks. The patient usually presented confabulation and visual and auditory hallucinations [2]. Other symptoms range from abnormal eye movements, motor incoordination, hypotension, elevated heart rate, and muscle weakness [1, 2]. The incidence of KS is higher in elderly subjects, and it is more prevalent in males. The literature related to neuropsychological and neurobehavioral alterations secondary to a diagnosis of Korsakoff's syndrome usually is insufficient; however, there are studies that reveal cognitive deficits in working memory and/or immediate verbal memory tasks.

The treatment for KS consists of improving the patient's quality of life (QOL) immediately, controlling the symptoms, and avoiding the evolution or worsening of the disease [3]. According to different studies [2, 3], the KS has an evolutionary and ultimately chronic process. Most of the patients who suffer from this disease usually end up with a clinical picture of Korsakoff's dementia, which is accompanied by neurobehavioral disorders, disinhibition, irritability, social isolation, hallucinations, delusions of persecution, and aberrant motor behavior.

Other studies [4, 5] demonstrate the existence of cognitive deficiencies in comparison with control subjects, alterations to plan and control immediate behavior (executive functions), inability to consolidate information containing new learning (working memory), and the storage and recall of long-term memory; the neuroanatomical structures that are usually involved are the frontal lobe of executive nature, the temporal lobe (for the consolidation and evocation of information), and diencephalic structures such as the amygdala, hippocampus, hypothalamus, and fronto-subcortical circuits [4]. Other findings [6] demonstrate alterations in the execution of visuospatial and visuoconstructive tasks, language, emotional area, and personality [7]. The aim of this work is to investigate the neuropsychiatric alterations presented by the patient with a diagnosis of advanced Korsakoff's syndrome.

## 2. Case Report

The case of a 63-year-old male patient, married, right-handed, occupation: industrial engineer, with higher education is reported. The patient presented with recurrent memory loss without loss of consciousness, accompanied by a mixed clinical picture of depression with a greater prevalence of anxiety.

The clinical history reveals an antecedent of alcohol abuse since the age of 16, related to a clinical evolution with apparent neurological deficit, aware, and oriented but with low verbal fluency. The general examination showed no evident alterations, and his vital signs were normal, blood pressure 120/80 mmHg, pulse 80, and respiratory rate 20. The patient takes losartan (every 12 hours) to treat his blood pressure and vitamin B1 (thiamine) to regulate his body.

In the neurological examination, the patient was conscious, aware, oriented in his individual, temporal and spatial sphere/dimensions, but with low verbal fluency, mild alterations in gait, difficulty in taking a sequence of steps, and clinical bradypsychia.

**Figure 1:**
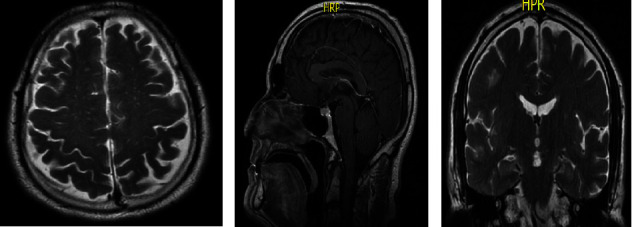
Results of MRI at cortical (a), sagittal (b), and coronal sections at the level of the temporal lobe (c) showing widening of cerebral sulci, subcortical atrophy, and hippocampal atrophy in the patient with advanced Korsakoff's syndrome.

The MRI revealed widening of cerebral sulci at a cortical level (Figure 1(a)) volumetric reduction of brain tissue and involvement of superior frontal gyrus. In the sagittal section is observed atrophy in the marginal callosal fissure, widening of the paracentral sulcus, and atrophy in the superior frontal gyrus (seen from the medial side) (Figure 1(b)). The image at the coronal slice (Figure 1(c)) revealed another temporal lobe involvement, especially in the lateral fissure or Sylvian fissure, with the left hippocampal atrophy involvement which is related to a deficit in consolidation processes and information storage.

We have a neuropsychology clinic protocol with the neuropsychological tests for the evaluation of the patient (Table 1). The direct score obtained in each of the tests has been considered, except for the DRS, in which the total score and the values of each of its subsections were considered.  Table 2 shows the results of each of the neuropsychological tests.

## 3. Discussion

The patient does not present clinical signs of depression. The test score shows that there is no presence of low emotional reactivity; however, it has revealed a clinical picture of generalized anxiety. The test results indicate that the patient presents a clinical picture of moderate-severe psychosocial stress, associated with movement, impatience, and motor fluctuations at the time of evaluation. The patient is partially oriented in time, place, and space. The results of the MMSE reveal alterations in memory tasks, inhibition tasks, and visuospatial functions. The results are not within the expected range.

**Figure 2:**
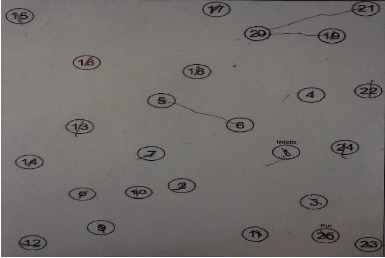
Trail making test (TMT) form A results. Duration: 3′52″.

During the evaluation of the attentional functions, in the DRS and TMT's sections that evaluate attention tasks, the patient showed not being able to maintain the attentional focus (focused attention) on each of the stimuli presented (Figure 2).

**Figure 3:**
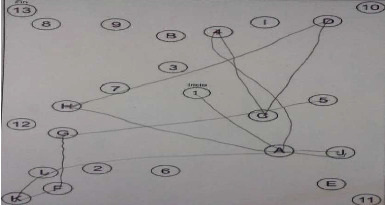
Trail making test (TMT) form B results. Duration: 3′47″.

It has been identified as a difficulty for a behavioral response during the test; additionally, it has found the inability to process several stimuli at the same time; in this case, the patient cannot generate a response to one stimulus in the presence of others (TMT part A and B) (selective attention) (Figure 3). Similarly, there was no evidence of cognitive interferences that would impede the performance of the tests. These data show that the patient could present alterations in brain areas that are usually related to attention, especially in working memory tasks, generally correlated with the frontal lobe.

The patient's memory has been assessed using the digit retention test, Babcock's story recall test, clock-drawing test, Rey's complex figure test, and an immediate and delayed verbal memory task. During the evaluation, the patient presented severe difficulty to process immediate information; this has been revealed by the use of material that stimulates short-term memory; additionally, he showed to not be able to retain new information or generate new learning. The digit retention test revealed a very low score; therefore, the patient could present an inability to manipulate information in situations which require quick decisions.

The patient is not able to remember the information immediately given. This type of memory has been evaluated by Babcock's story recall test. The test revealed that the patient presents difficulty to explain the verbal material presented by the auditory pathway; moreover, he did not present the cognitive skills necessary to register, encode, consolidate, and recover the information previously given. Similarly, in Rey's complex figure test, a test was presented visually; the patient demonstrates to be unable to complete the figure presented on the test; then, in the memory test, he could not perform it. These results suggest a serious difficulty related to short- and long-term memory in tasks of visuospatial functioning and visuoconstructive skills. Despite the results of the copy, the patient defines some details, but it is not enough for the expected range for his age.

**Figure 4:**
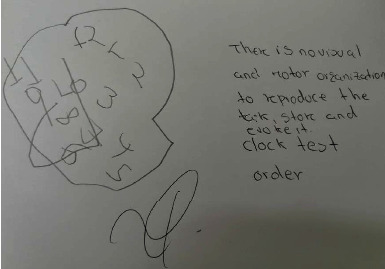
Clock test on command.

These results are confirmed by the DRS's memory subsections, which revealed the lack of skills or cognitive strategies in the patient to process, consolidate, and recall information. The scores obtained are below the expected. Similarly, in the memory test, a low score has been obtained, which confirms an inability in working memory tasks and short- and long-term memory tasks, thereby, preventing the preservation of the mnesics content and dysfunction of brain structures associated with memory such as the neurocognitive domain. In the clock-drawing test, it was also identified (in command and copy) that there is no visual and motor organization to reproduce, store, and recall the task (Figure 4).

From executive functions results, it was found that the patient presents an inability to plan, organize, and regulate his immediate behavior. The DRS revealed that the patient lacks motor, cognitive, and behavioral functions to perform activities that require intentionality, control, and behavior in the execution of a task, the same situation is for the FAB; although the score is very low and may denote a clinical picture of dementia, because of his age and the presence of KS, it cannot be delimited in this way, a similar situation is for the DRS. The explanation for this data is that the patient´s disease is still evolving, generating a clinical picture of cognitive deficit, which is represented by a cortico-subcortical atrophy, that affects the cognitive domains previously explained. In this case, the domain affected is verbal fluency because the patient did not present the mental and oral flexibility to generate common names, animals, and both in an alternating state. These data are possibly related to the dysfunction in his frontal lobe and dorsolateral cortex.

Regarding the basic and instrumental activities of daily living, the Barthel index revealed that the patient is independent; nevertheless, these data have not been confirmed by the Lawton and Brody scale, which revealed that the patient is entering a phase of dependence that needs to be assessed immediately, since it may be an indicator of progressive cognitive impairment, which as the disease evolves, may be reflected in a clinical picture of dementia.

## 4. Conclusion

The results indicate that the patient presents neuropsychological alterations in neurocognitive domains, such as attention, because the patient is not able to retain the attentional focus, or to manipulate the information when presented with different stimuli [6].

The data obtained by different studies [4–6] indicate that patients with KS usually present severe problems in the storage, processing, consolidation, and evocation of information, as occurs with the case report. The patient showed difficulty generating new memories; his mental processes in relation to brain structures in the storage of information do not allow the condensation and recovery correlated with the immediate operative and/or verbal memory.

Also, the deferred verbal memory showed to be altered [6] because the patient demonstrates to be unable to access previously learned memories, a situation that usually gets worse during the evolution of the disease; therefore, it requires immediate and multidisciplinary attention.

In executive functioning tasks, it has been found that serious difficulties regulate and control the immediate behavior; the age and KS that the patient suffers are directly correlated with the cortico-subcortical atrophy that he is presenting, which causes a lower performance in tasks that require cognitive flexibility, conceptualization, and the definition of clear goals or objectives [5, 6]. Unfortunately, the patient showed to be unable to generate a behavioral pattern that is accompanied by a new response, and against a variety of stimuli, his low verbal fluency demonstrates the inability to have executive control, anticipation, action, and planning to perform certain motor behaviors having a clear and specific purpose. These alterations may be related to a dysexecutive syndrome [6, 7]. These neuropsychological deficits are correlated with frontal hypometabolism in positron emission tomography (PET) [8].

Regarding the activities of daily living, the patient is moderately independent; however, by making errors such as confuse the denomination of money and forget the consumption of his medications, suggest and even affirm, that in a short time, the clinical picture of the patient could progress to Korsakoff-type dementia, which is linked to the score he obtained in the GHQ-28. Currently, the patient presents a neuropsychiatric episode where he does not recognize his disease, accompanied by the chronicity of each of his symptoms, especially in anxiety and less in depression. For this reason, it is a necessary multidisciplinary intervention, which comprises specialties such as internal medicine, psychology, neuropsychology, neurology, physiotherapy, and occupational health, which have a clear objective, the improvement of his QOL [9–14].

Based on the results obtained, it can be inferred that the patient in the medium term will present a progressive neurocognitive deficit associated with a possible clinical picture of dementia due to KS. It is considered necessary to carry out prevention campaigns focused on Korsakoff's disease, with the purpose of preventing major neuropsychological alterations in patients with this type of diagnosis. Also, neurorehabilitation plans by expert neuropsychologists become a unique opportunity to improve the quality of life in patients with this disorder. Future lines of research should focus on functional neurorehabilitation plans aimed at the early diagnosis and treatment of this disease, accompanied by epidemiological studies focused on the study of the incidence and prevalence of this disease.

## Figures and Tables

**Table 1:** Assessment protocol that aims at neuropsychological tasks as a function of Korsakoff's syndrome in the patient.

Test	Description
Mini-mental test (MMSE)	It is a brief cognitive tracking test; it evaluates cognitive functions; it is composed of 30 sections and grouped into five dimensions: orientation (10 points), fixation (3 points), orientation (5 points), calculation and memory (3 points), language (8 points), and visuoconstructive skills (1 point)
Dementia rating scale (DRS-2)	It is a scale that aims to assess general cognitive aspects. It is an instrument with high validity for detecting clinical pictures of dementia. It has a total score of 144 points, grouped into 5 subsections: attention (37 points); initiative/perseveration (37 points); construction (6 points); conceptualization (39 points); memory (25 points)
Digit retention	Weschler scale digit retention: It is a subtest of the WAIS-IV that aims to measure the levels of attention and immediate verbal memory
Trail making test (TMT)	TMT-A: It is a paper and pencil test that aims to measure the levels of attention. The patient must complete the test in the shortest possible time.
	TMT-B: The subjects must match numbers from 1 to 25 consecutively, and executive functions which consists of matching numbers from 1 to 13, but alternating with letters (1A-2B-3C-4D-5D and so on consecutively). The patient must complete the test in the shortest possible time.
Babcock's story recall test	It aims to assess verbal and delayed memory. The test is presented by the auditory pathway. Rey's complex figure.
Rey complex figure	It is a screening test or brief cognitive tracking; its objective is to evaluate cognitive functions in patients with neurological or neurodegenerative diseases. It is a test that aims to assess visuospatial memory and visuomotor ability.
The clock-drawing test	It is a screening test or brief cognitive tracking; its objective is to evaluate cognitive functions in patients with neurological or neurodegenerative diseases. It is a test that aims to assess visuospatial memory and visuomotor ability.
Memory test	It is a test that aims to assess immediate and delayed verbal memory. A series of words is read to the patient; then, he must verbalize them. After a while, the patient is asked to remember the list of words.
Brief frontal assessment battery (FAB)	It is a specific test that measures executive functioning and cognitive deficits or low performance in older adults or those diagnosed with a neurodegenerative disease.
Verbal fluency	The patient presented with a task where he has to name animals and common names in one minute. Furthermore, it can also be performed alternately, when the subject must name a word, alternating it with a category required by the evaluator. The score is obtained by the sum of the correct answers.
Barthel index	It is a questionnaire that aims to evaluate and assess the level of functional independence of the patients, during basic activities of daily living.
Lawton and Brody	It is a questionnaire that assesses the level of functional independence of the subjects to perform instrumental activities of daily living.
Health self-perception questionnaire (GHQ-28)	It is a questionnaire that evaluates health and self-perception of health in subjects with different clinical conditions. The questionnaire is grouped into four sections of seven items (somatic symptoms, anxiety/insomnia, social dysfunction, and depression). A score greater than or equal to 23 points is a possible indicator of a psychiatric condition.

**Table 2:** Scores obtained in different neuropsychological tasks as a function of a patient with Korsakoff's syndrome.

Test		Highest score	Score obtained
Mini-mental test (MMSE)		30	22
Dementia rating scale (DRS-2)		144	91
Attention		37	27
Initiation/perseveration		37	20
Construction skills		6	6
Conceptualization		39	29
Memory		25	9

Digit retention	Direct	8	6
	Inverse	7	0

Trail making test (TMT)	TMT-A		3′52″
	TMT-B		3′47″

Babcock's story recall test	Immediate recall	21	7
	Delayed recall	21	3

Rey complex figure	Copy	36	2′47″
	Memory	36	Cannot do it

The clock-drawing test	Copy/command	10	7
		10	3

Memory test		12	3
Brief frontal assessment battery (FAB)		18	8

Verbal fluency	Animals		4
	People		3
	Alternant		Cannot do it

Barthel index		100	100
Lawton and Brody		7	5
Health self-perception questionnaire (GHQ-28)		84	48

## Data Availability

The neuroimages data and test applied during the assessment that support the findings of this study are included within the article. Any other data related to this research are available from the corresponding author on reasonable request.
